# Endometrial Stem Cells: Orchestrating Dynamic Regeneration of Endometrium and Their Implications in Diverse Endometrial Disorders

**DOI:** 10.7150/ijbs.89795

**Published:** 2024-01-01

**Authors:** In-Sun Hong

**Affiliations:** 1 Department of Health Sciences and Technology, GAIHST, Gachon University, Incheon, 21999, Republic of Korea.; 2 Department of Molecular Medicine, School of Medicine, Gachon University, Incheon 406-840, Republic of Korea.

## Abstract

The human endometrium, a vital component of the uterus, undergoes dynamic changes during the menstrual cycle to create a receptive environment for embryo implantation. Its remarkable regenerative capacity can be attributed to the presence of tissue-resident stem cell populations within the endometrium. Despite variations in characteristics among different subtypes, endometrial stem cells exhibit notably robust self-renewal capacity and the ability to differentiate into multiple lineages. This review offers a comprehensive insight into the current literature and recent advancements regarding the roles of various endometrial stem cell types during dynamic regeneration of the endometrium during the menstrual cycle. In addition, emerging evidence suggests that dysfunction or depletion of endometrial stem cells may play critical roles in the development and progression of various endometrial disorders, such as endometriosis, uterine fibroids, adenomyosis, infertility, and endometrial cancer. Therefore, we also highlight potential roles of endometrial stem cells in the development and progression of these endometrial diseases, including their ability to accumulate genetic mutations and express genes associated with endometrial diseases. Understanding the dynamic properties of the endometrium and the roles of endometrial stem cells in various endometrial disorders will shed light on potential therapeutic strategies for managing these conditions and improving women's fertility outcomes.

## Introduction

The human endometrium, a highly dynamic tissue inner lining the uterine cavity, undergoes a cyclic regeneration and shedding approximately 400 to 500 times during menstrual cycles, all meticulously orchestrated under the precise regulation of steroid hormones, specifically estrogen and progesterone 1. This cyclical renewal process is essential for creating a receptive environment for embryo implantation during pregnancy or facilitating physiological menstruation in the absence of fertilization. During each menstrual cycle, it can regenerate and grow rapidly, increasing in thickness by approximately 4-10 mm within 1 week 2. At the core of this extraordinary regenerative capacity lies a small population of endometrial stem cells, which play a pivotal role in orchestrating the regenerative process ensures the continuous flourishing of this dynamic tissue 3. Endometrial stem cells represent a unique subset of cells within the endometrial tissue, possessing the remarkable ability to self-renew and differentiate into various cell types, including stromal, epithelial, and vascular cells. In the proliferative phase of the menstrual cycle, the endometrial stem cells respond to hormonal signals, particularly estrogen, which initiates a cascade of molecular events driving endometrial proliferation. These newly generated cells contribute to the thickening of the endometrial lining, fostering a supportive environment for potential embryo implantation.

In 1978, Prianishnikov was the first to propose the presence of indigenous pluripotent stem cells within the endometrial tissue, owing to its remarkable regenerative capabilities 4. In 2004, a pioneering study performed by Chan et al. marked a significant milestone as it successfully identified and comprehensively characterized clonogenic small human endometrial stem cell subpopulations for the very first time 5, 6. They identified that about 1.2% of stromal-like cell populations and approximately 0.2% of epithelial-like cell populations exhibited high proliferative potential and remarkable ability to differentiate into diverse cell types 5. Currently, the accumulations of endometrial stem cell abnormalities and mutations may play a pivotal role in the onset and advancement of diverse endometrial diseases such as endometrial cancers, endometriosis, and thin endometrium-associated infertility 7. For example, thin endometrium stands out as a significant causative factor linked to endometrial receptivity and subsequent lower pregnancy success rates. Indeed, Tewary et al. reported their findings, noting a correlation between diminished clonogenic endometrial cell populations at the baseline of endometrium and the comparative severity of recurrent pregnancy loss arising from impaired endometrial growth 8. Furthermore, endometrial stem cells stand out as exceptional sources among adult stem cells, characterized by their robust multipotency, easy accessibility, high yield, and adherence to ethical standards, rendering them versatile for both autologous and allogeneic applications 9. For example, the transplantation of human endometrial stem cells has shown remarkable acceleration of injured endometrial tissue regeneration, primarily attributed to their ability to enhance angiogenesis and increase the thickness of endometrial functional layer 10.

Until now, two hypotheses have been proposed regarding the origin of uterine stem cells: the endogenous origin hypothesis posits their location in the basal region of the endometrium, while the exogenous origin hypothesis suggests they originate from bone marrow stem cells 9. For example, stromal-type endometrial stem cells have been observed in proximity to the luminal and glandular epithelia in both the functional and basal layers of the endometrium. This observation implies that while certain stem cell subpopulations in the functional layer may be shed during menstruation, specific subpopulations situated in the basal layer are likely to persist 11-13. Conversely, Taylor et al identified donor-derived endometrial cells in biopsy samples of human endometrial tissue collected from recipients of bone marrow stem cells. These cells constituted approximately 52% of the endometrial stromal cell populations and 48% of the epithelial cell populations. This finding implies that bone marrow-derived stem cells could serve as an external source of endometrial cells 6.

This review paper aims to describe the dynamic properties of the human endometrium, with a particular focus on the roles of various endometrial stem cell types in in the process of tissue regeneration. Through a comprehensive review of existing literature and recent advancements, we will provide a concise overview of how the dysregulation of endometrial stem cells might play a contributory role in diverse endometrial disorders. Understanding these mechanisms is crucial for advancing our knowledge of female reproductive health and developing potential therapeutic approaches to manage endometrial-related pathologies.

## Dynamic properties of human endometrium

The human uterus is vital for facilitating gestation, labor, and delivery. Within the uterus, the endometrium, which serves as the innermost mucosal lining, is responsible for attachment and implantation of the embryo 14. Endometrial glands play a crucial role in providing initial nutrition to the embryo until the placenta develops 15. For successful attachment, implantation, and placentation to occur, effective communication between implantation-competent embryo and receptive endometrium must take place in a timely and efficient manner 16. Proper functioning of the endometrium is reliant upon luminal and glandular epithelia, which are composed of both ciliated and secretory cells. These two types of cells work in conjunction to carry out a range of vital functions. The luminal epithelium located on the surface of the endometrium serves as the site of attachment for the embryo during implantation 17. Meanwhile, the glandular epithelium consists of long tubular glands that release secretions containing a variety of growth factors and lipids essential for placental development 18. It is important to note that secretory cells within the glandular epithelium play a crucial role in creating the environment necessary for successful implantation and pregnancy. Secretions produced by these cells not only provide nutrients needed for the developing embryo, but also regulate the growth and differentiation of trophoblast cells, which are responsible for forming the placenta 19.

The development of the endometrium is a complex and finely orchestrated process that begins during embryonic and fetal development. It commences with the formation and elongation of the müllerian ducts, precursors to the female reproductive tract 20. The müllerian ducts differentiate into the uterine tubes, uterus, and cervix through intricate interactions involving signaling pathways like Wnt and Retinoic Acid, as well as the regulation of homeobox genes such as HOXA and HOXD 21. As the uterine rudiment takes shape, epithelial-mesenchymal interactions become pivotal in the development of glandular structures within the endometrium 22. Hormonal regulation, particularly by estrogen and progesterone, influences the proliferation and differentiation of endometrial cells, mirroring the morphological characteristics of the adult endometrium even during fetal development 23, 24. The extracellular matrix undergoes significant remodeling, with fibronectin, collagens, and proteoglycans establishing the structural foundation, and integrins mediating cell-ECM interactions 25. Vascularization is a critical aspect of endometrial development, driven by angiogenic factors like VEGF and angiopoietins 26. Bone-marrow-derived endothelial progenitor cells contribute to the formation of blood vessels, ensuring proper nutrient supply and tissue perfusion 27. The clinical significance of understanding endometrial development lies in its implications for congenital reproductive tract abnormalities 28 and conditions like müllerian anomalies 29. Insights gained from developmental processes may offer novel therapeutic strategies for addressing reproductive health challenges related to endometrial development.

**Figure 1:**
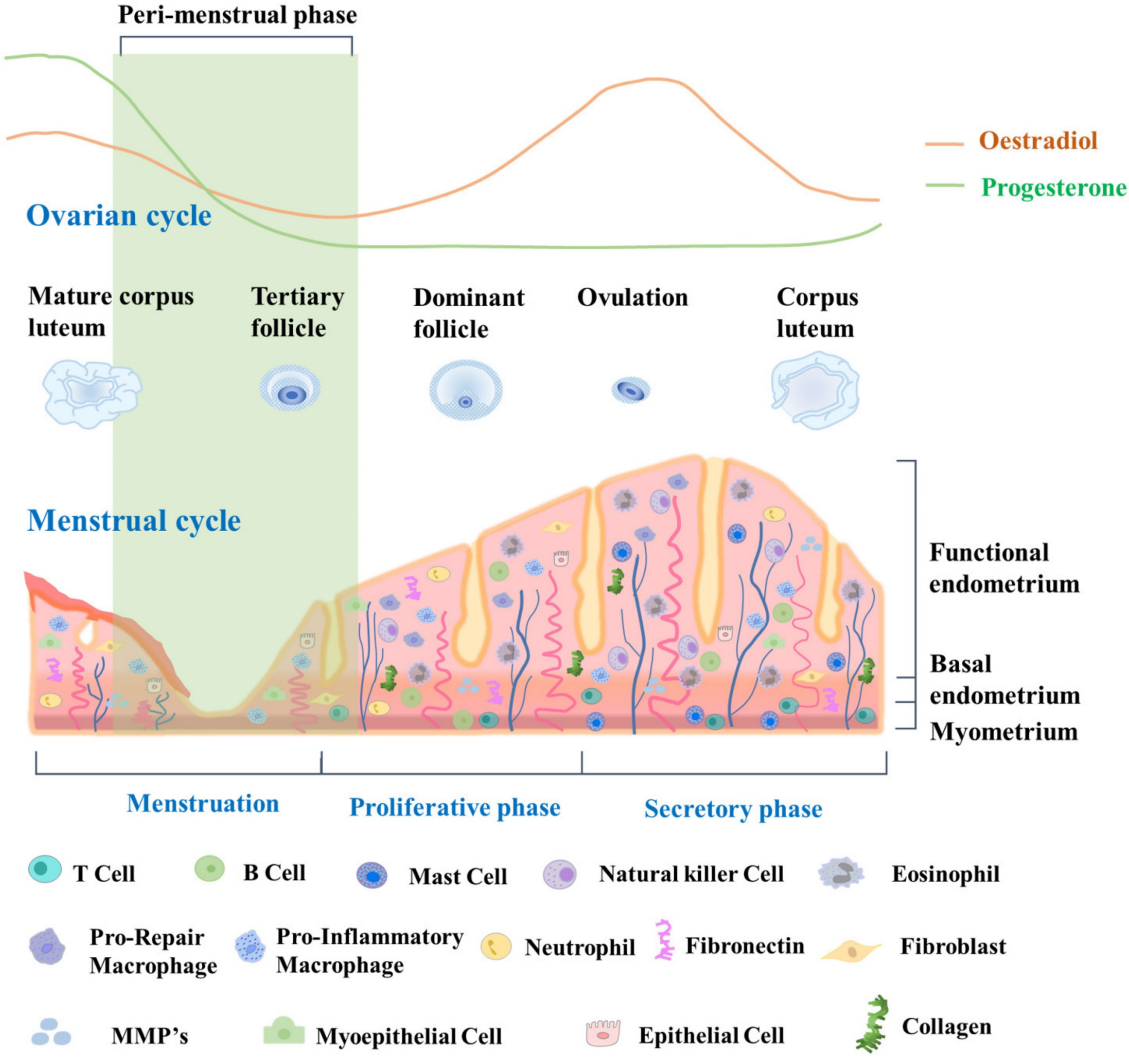
** Dynamic changes in endometrial tissue throughout the menstrual cycle and its constituent cells.** During the proliferative phase, the endometrium exhibits increased glandular and stromal cell proliferation, preparing for potential implantation. The secretory phase demonstrates enhanced glandular secretion and development of a rich vascular network, indicating a favorable environment for embryo implantation. Epithelial cells line the luminal endometrium, forming the luminal epithelium. They regulate the secretion and absorption of substances, facilitating embryo implantation. Glandular cells secrete substances such as glycogen, lipids, and proteins. They create an optimal environment for embryo implantation and provide nourishment. Vascular cells, including endothelial cells, form blood vessels that supply oxygen and nutrients to the endometrium. Endometrial stem cells are a population of cells that reside in the endometrium and possess the ability to self-renew and differentiate into various cell types. These stem cells contribute to the regenerative process of the endometrium, allowing for the renewal and repair of the tissue after menstrual shedding.

The endometrial cycle is intricately linked to the ovulation cycle. Its three distinct phases work together to create a receptive environment for a potential pregnancy. By coordinating with the three phases of the ovulation cycle, the endometrial cycle ensures that the uterus is adequately prepared to support a developing embryo if fertilization occurs 30. During the proliferative phase, which occurs in the first half of the cycle, the endometrium undergoes extensive growth and thickening driven by the action of estrogen on the endometrial epithelium and stroma. Epithelial and stromal cells undergo proliferation and differentiation, giving rise to glandular structures and a complex extracellular matrix 31. In the second half of the cycle, which is the secretory phase, the endometrium continues to thicken in preparation for potential implantation of a fertilized egg. The secretory phase is driven by the action of progesterone, which promotes differentiation of the endometrial epithelium and stroma, leading to the formation of highly specialized structures that can support early pregnancy 32. These structures include secretory glands, decidualized stroma, and a specialized extracellular matrix. If implantation does not occur, the endometrium undergoes programmed cell death and shedding during the menstrual phase, which marks the beginning of a new menstrual cycle. This shedding is facilitated by decreases of estrogen and progesterone levels, which then trigger the onset of menstruation 33. Due to its dynamic structural changes during the menstrual cycle, endometrial tissue is an excellent model for studying tissue regeneration and repair, particularly with regard to stem cell research. Fig. 1 provides a detailed description of the dynamic changes occurring in the endometrium throughout the menstrual cycle.

## Roles of various types of endometrial stem cells in endometrial regeneration

Recent studies have revealed that the dynamic endometrium is a rich source of several types of tissue resident stem cells 34, 35. Endometrial stem cells are a subpopulation of cells with the capacity for self-renewal and differentiation into multiple cell types that compose the endometrium 36, 37. Indeed, endometrial tissue contains various types of stem cells, including epithelial-like stem cells 38, stromal-like stem cells 39, and perivascular endometrial stem cells 40. Each of these populations has unique molecular and functional characteristics. During the menstrual cycle, endometrial stem cells are activated in response to hormonal cues from the ovary 41. This process is tightly regulated by a complex interplay of hormonal signaling and cellular processes, including cell adhesion, proliferation, differentiation, and apoptosis 39, 42, 43. Such activation leads to regeneration of the endometrium, with proliferation and differentiation of stem cells into various cell types that compose the endometrial lining 42. These stem cells have been shown to have the potential to differentiate into a variety of cell types, including luminal epithelial cells, smooth muscle cells, fibroblasts, and blood vessel cells 41. Although the precise mechanisms of stem cell activation and differentiation are not yet fully understood, they are thought to involve complex interactions between various signaling pathways and transcription factors. Understanding these complex interactions and the role of endometrial stem cells during the dynamic menstrual cycle could lead to the development of new therapies for various conditions that affect endometrial tissue regeneration, such as infertility, endometriosis, and endometrial cancer.

### Epithelial-like endometrial stem cells

**Figure 2:**
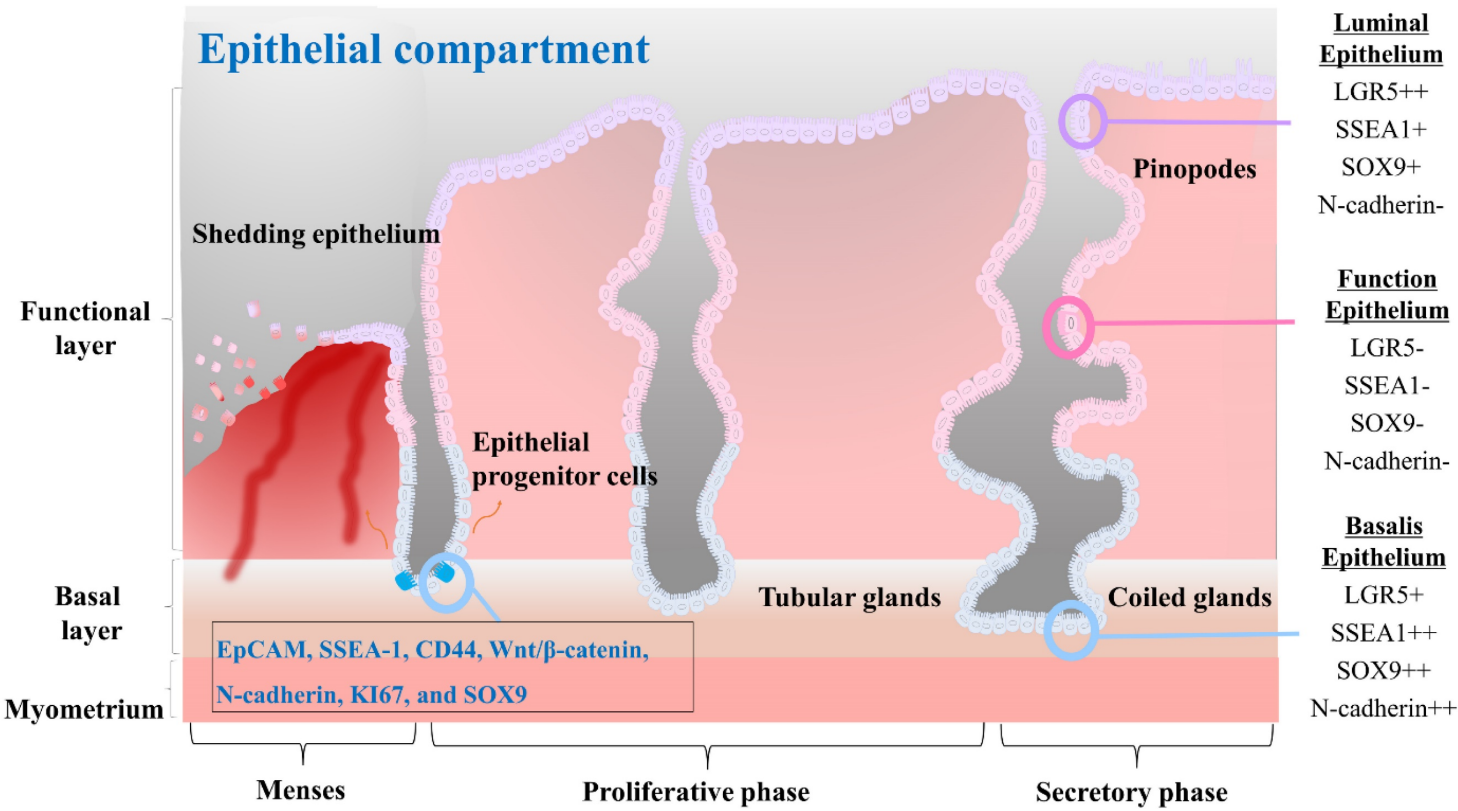
**The characteristics of the three different cells that constitute the endometrial epithelial compartments and the endometrial progenitor cells.** The luminal epithelium represents the surface layer of the endometrium. It plays a vital role in facilitating embryo implantation and establishing communication between the embryo and the endometrium. These cells commonly express surface proteins such as LGR5, SSEA1, SOX9, αvβ3 integrin, and MUC1. The functionalis epithelium undergoes cyclical changes in response to hormonal fluctuations and is shed during menstruation. It creates an optimal environment for embryo implantation and subsequent pregnancy. They often express surface proteins such as estrogen receptors (ER) and progesterone receptors (PR), which mediate hormonal signaling and regulate the receptivity and secretory functions of the endometrium. The basalis epithelium forms the basal layer of the endometrial epithelium. It remains relatively unchanged throughout the menstrual cycle and provides a source of regenerative cells for the renewal and repair of the endometrium. Basalis epithelial cells may express surface proteins involved in cell proliferation and regeneration, such as Ki67, CD146, LGR5, SSEA1, SOX9, and N-cadherin. Endometrial progenitor cells possess self-renewal and differentiation capabilities, contributing to the replenishment of the luminal and functionalis epithelial cells. Surface markers for them may include EpCAM, SSEA-1, CD44, Wnt/β-catenin, N-cadherin, KI67, and SOX9, among others, depending on the specific subpopulation of progenitor cells.

Epithelial stem cells in the endometrium are thought to be located at the base of the glands in the basalis layer 44-46. These stem cells are capable of both self-renewal and differentiation, allowing them to give rise to various epithelial cell types of the endometrium. They are also responsible for generating luminal epithelial cells that line the endometrial cavity. Initially, endometrial epithelial stem cells were identified by Chan et al. as highly clonogenic cells, accounting for only 0.22% of single cell subpopulations of EpCAM positive epithelial cells derived from hysterectomy endometrial tissue containing the basalis layer 5. *In vitro* culturing of epithelial stem cells for prolonged durations have proven challenging as the *in vivo* phenotype of these cells is not maintained using standard monolayer culture methods. Recent studies have highlighted differences in the presence of stage-specific embryonic antigen-1 (SSEA-1) positive cells in the epithelium of the endometrium during different stages of the menstrual cycle. Specifically, a greater number of SSEA-1 positive cells have been observed in the epithelium of the proliferative phase compared to the secretory phase, with a greater number of these cells present in the basalis than in the functionalis epithelium 47, 48. Moreover, these SSEA-1 positive cells have demonstrated progenitor activity in short-term *in vitro* monolayer culture, leading to the presumption that they represent a population of epithelial stem cells 44. A long-term culture condition has been developed by He et al. utilizing a combination of small molecules to enable *in vitro* culture and expansion of human SSEA-1 positive epithelial stem cells 38. In addition, their research revealed that SSEA-1 positive cells exhibited greater therapeutic potential for intrauterine adhesion than endometrial mesenchymal/stromal stem cells. In fact, *in situ* injection of SSEA-1 positive cells-laden chitosan into an animal model with intrauterine adhesion resulted in effective reduction of fibrosis and facilitation of endometrial regeneration 38. Epithelial stem cells are regulated by a complex interplay of hormonal and paracrine signals, including signals from the surrounding stroma and immune cells. For example, Janzen et al. have found that estrogen and progesterone can regulate the proliferation and differentiation of CD44/EpCAM positive epithelial stem cells as well as Wnt/β-catenin pathway and its downstream target genes such as Axin2, CD44, c-Myc, and ID2 49. Several markers that are characteristic of endometrial epithelial stem cells, including N-cadherin, KI67, and SOX9, have been identified. These cells also express markers such as WNT, LGR5, and PAX8 50. The characteristics of the different epithelial cells found in each compartment of the endometrial tissue, as well as the molecular features of the epithelial progenitor cells, are properly illustrated in Fig. 2.

### Stromal-like endometrial stem cells

**Figure 3:**
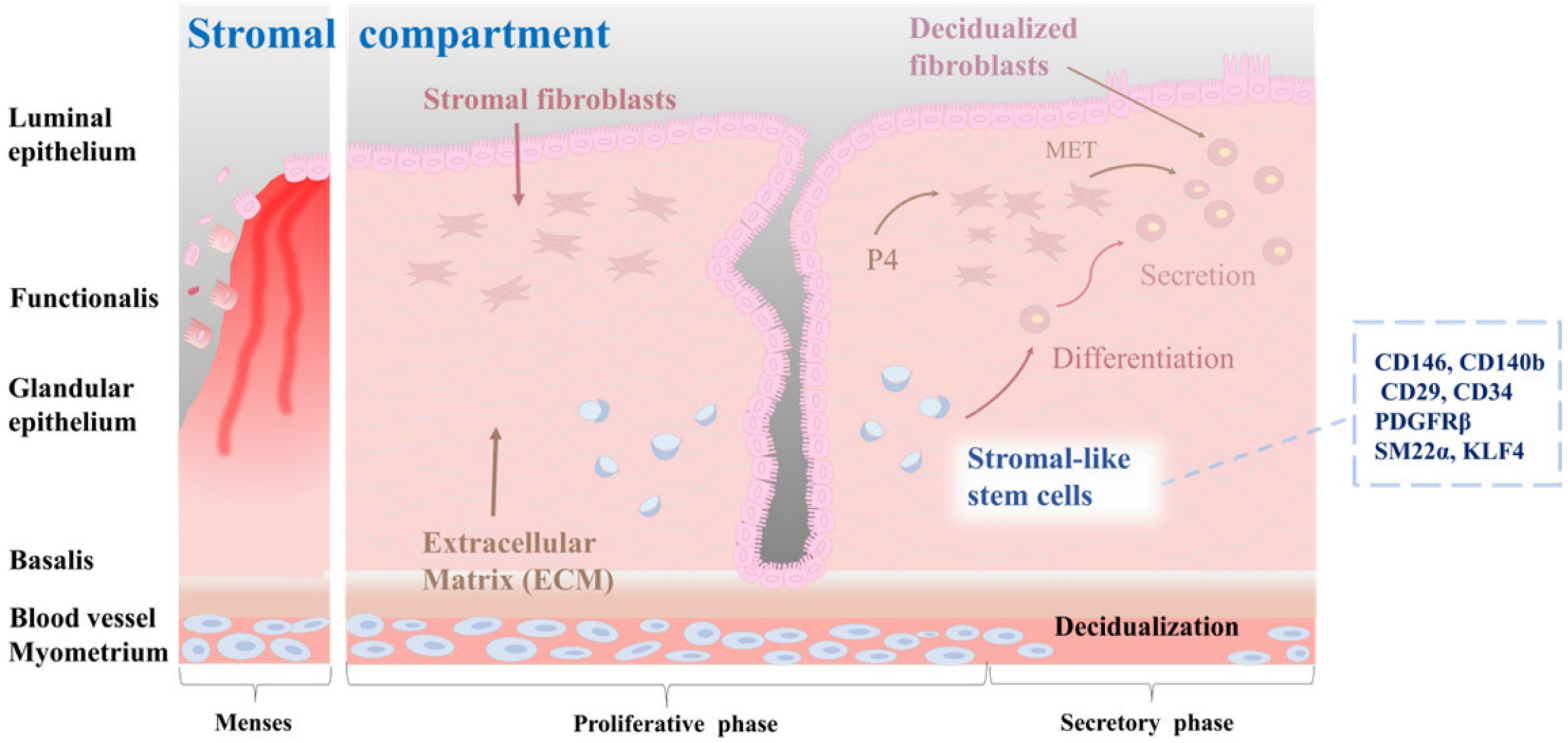
** Characterization of stromal-like stem cells, and their differentiation into decidualized stromal cells.** The endometrial stromal compartment plays a critical role in supporting embryo implantation, vascularization, and tissue remodeling during the menstrual cycle. Stromal-like stem cells are a distinct population within the endometrial stromal compartment. These cells possess self-renewal and multilineage differentiation capabilities, contributing to the dynamic nature of the endometrium. Stromal-like stem cells can differentiate into decidualized stromal cells. Decidualization is a unique transformation that occurs during the implantation process, involving changes in cell morphology and function to support embryo development, vascular remodeling, and immune modulation in the maternal-fetal interface. They also create a nurturing environment for embryonic implantation and early placental development.

Stromal stem cells in the endometrium are thought to be located in the perivascular region of the stroma, which is the connective tissue that supports endometrial glands and blood vessels 46. They are capable of differentiating into multiple cell types, including smooth muscle cells, fibroblasts, and endothelial cells 51. During the proliferative phase, stromal stem cells undergo extensive proliferation and differentiation, giving rise to stromal cells that support the endometrial epithelium 41. These cells are involved in the production of extracellular matrix proteins, growth factors, and cytokines that are essential for proper functioning of the endometrium 52. In addition, they are responsible for remodeling the endometrial vasculature necessary for the establishment of a receptive environment for embryo implantation 53. Stromal stem cells are regulated by a complex interplay of hormonal and paracrine signals, including signals from the surrounding epithelium and immune cells 54. For example, estrogen and progesterone can regulate the differentiation of stromal stem cells into decidualized cells by activating active DNA methyltransferases 55. Furthermore, stromal stem cells have immunomodulatory properties, which might be important for the regulation of immune responses in the endometrium. According to a study of Leñero et al. 52, therapeutic effects of CD146^+^ endometrial stromal cells are primarily mediated by secretion of a mixture of hsa-miR-320e, hsa-miR-182-3p, hsa-miR-378g, and hsa-let-7e-5p enriched factors. These factors target the immune system and modulate angiogenesis by regulating polarization of macrophages, activation of T cells, and transcriptional regulation of inflammatory cytokines such as TNF-α, IL-1β, and IL-6. Several markers that are characteristic of endometrial stromal stem cells, including CD140b, CD146, CD29, platelet-derived growth factor receptor beta (PDGFRβ), and sushi domain containing-2 (SUSD2, also known as W5C5), have been identified 56. Queckbörner et al. have isolated stromal endometrial stem cells from healthy donors during the proliferative stage of the menstrual cycle and shown that these cells exhibit an MSC surface phenotype characterized by the expression of CD90, CD73, CD105, CD45, CD34, CD14, CD19, HLA I, and HLA II. Additionally, these cells demonstrate multipotent differentiation capacity into adipocytes and osteoblasts 57. Recent studies have emphasized the significance of stromal-like mesenchymal stem cells in the regulation of immune responses, especially regulatory T cells. Aleahmad et al. have discovered that stromal endometrial stem cells contribute to the regulation of the uterine immune system by inducing functionally active CD4+ regulatory T cells through secretion of cytokines such as IL-6, IL-10, TGF-β, and IDO 58. Yin et al. have discovered a subset of SM22α^+^-derived CD34^+^KLF4^+^ stem or progenitor cells located in the endometrial stroma. When activated, these cells proliferate rapidly and migrate to the damaged epithelial area where they contribute to the process of endometrial regeneration. This regeneration process is correlated with increased protein SUMOylation observed in CD34^+^KLF4^+^ cells 59. The characteristics of the stromal-like endometrial stem cells within endometrial tissue, as well as their differentiation into decidualized cells, are properly illustrated in Fig. 3.

### Perivascular endometrial stem cells

**Figure 4:**
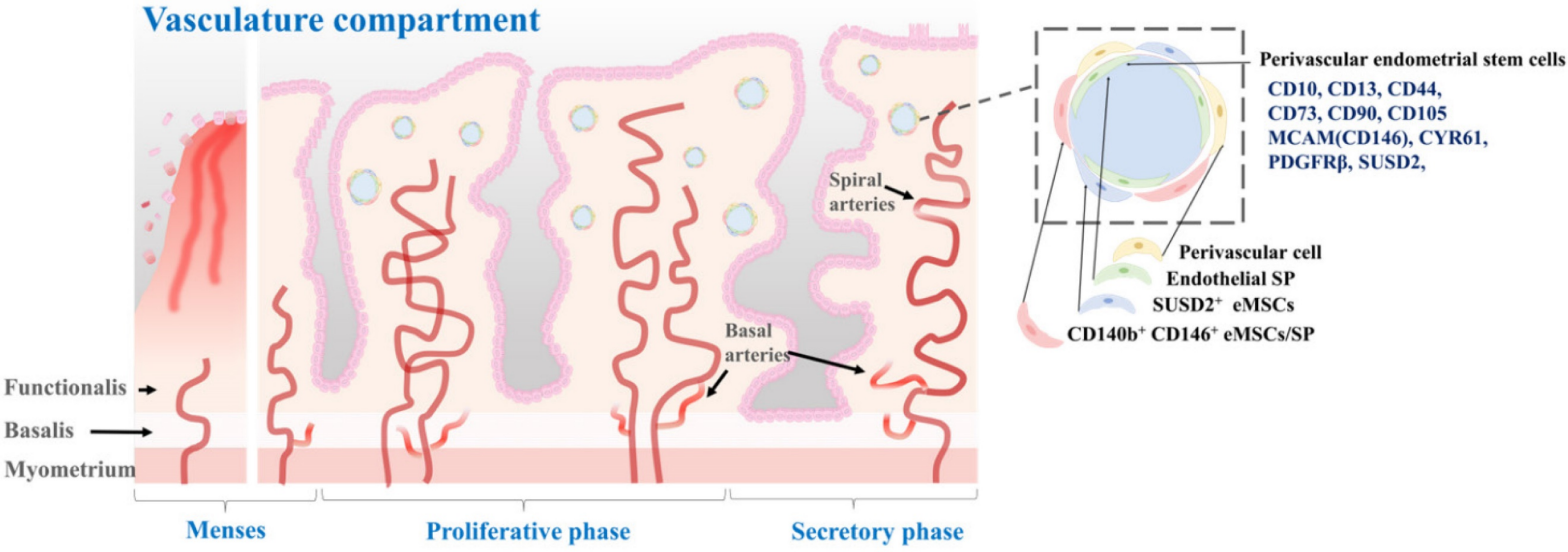
** Characterization of perivascular endometrial stem cells and their location within** the **vascular compartment of the endometrium.** The vascular compartment plays a crucial role in endometrial function by providing the necessary blood supply to support the dynamic changes during the menstrual cycle and early pregnancy. Perivascular endometrial stem cells are a specialized population of stem cells that reside in close proximity to the blood vessels within the endometrium. These stem cells possess unique regenerative and immunomodulatory properties. These stem cells have the capacity for self-renewal and differentiation, allowing them to contribute to tissue repair, regeneration, and angiogenesis. They also exhibit immunomodulatory properties and can interact with immune cells, influencing the local immune response and fostering an immunotolerant environment during pregnancy. Perivascular endometrial stem cells commonly express specific surface markers, including CD10, CD13, CD44, CD73, CD90, CD105 MCAM(CD146), CYR61, PDGFRβ, and SUSD2.

According to a previous study, pericytes, also referred to as Rouget cells or mural cells, are contractile cells that wrap around capillaries and microvessels. They play a key role in regulating blood pressure and angiogenesis 60. Pericytes also contribute to the formation of fibroblast-like cells and deposition of extracellular matrix, which can hinder axonal regeneration in cases of spinal cord injuries 61. Additionally, pericytes share several phenotypes and expressed genes with mesenchymal stem cells, leading to their consideration as the origin of these stem cells 62. Isolated as CD146^+^/CD34^-^/CD45^-^/CD56^-^ cells using flow cytometry across various organs, pericytes are considered a potential source of MSCs, owing to the numerous shared morphologies and gene expression profiles between the two cell types 63. Initially, pericytes exhibit comparable characteristics and phenotypes to MSCs, and under *in vitro* conditions, they can be differentiated into cells resembling fibroblasts. Secondly, both cell types express the conventional MSC biomarkers CD29, CD44, CD105, CD73, and CD90, while lacking the expression of hematopoietic stem cell biomarkers including CD45 and human leukocyte antigen-antigen D-related (HLA-DR). Thirdly, pericytes expressing CD146 can be prompted to undergo differentiation into various mesodermal lineages, including neural-like cells, adipocytes, and osteoblasts. Perivascular stem cells are located in the perivascular space of the endometrium. They can differentiate into both epithelial and stromal cell types 62, 64. They are thought to play a role in the formation and maintenance of endometrial vasculature. These cells are located in the perivascular region of the endometrium, in close proximity to blood vessels. They are characterized by the expression of specific cell surface markers such as CD146, PDGFRβ, and SUSD2 65. One of the main roles of perivascular stem cells is to give rise to the stromal compartment of the endometrium, which is essential for proper functioning of the endometrium during the menstrual cycle 57. Spitzer and colleagues have isolated perivascular endometrial stem cells that expressed both MCAM (CD146) and PDGFRβ from patients undergoing benign gynecologic surgery 64. They found that these stem cells were highly purified with clonogenic potential. These cells were multipotent. They were located in the perivascular region of the adult human endometrium. In addition, gene expression profiling results indicated that these perivascular endometrial stem cells had a phenotype that was consistent with self-renewal, multipotency, and immunomodulation 64. Fan et al. have isolated SUSD2^+^ perivascular endometrial stem cells from patients with recurrent implantation failure who have undergone two sequential local endometrial injury. They found that SUSD2^+^ perivascular endometrial stem cells were clonogenic, highly proliferative, and capable of decidualization. Although the clonogenicity and proportion of SUSD2^+^ cells did not change after a local endometrial injury, there was a trend towards a higher proliferation rate with shorter doubling time during the second endometrial injury. However, the degree of SUSD2^+^ perivascular endometrial stem cell decidualization was significantly reduced in the second injured endometrial biopsy compared to that in the first injured biopsy 40. Li et al. have sorted human perivascular endometrial stem cells by flow cytometry using CD10, CD13, CD44, CD73, CD90, and CD105 antibodies and demonstrated their capability to differentiate into adipocytes, osteoblasts, and neuron-like cells. Additionally, they observed that transplantation of perivascular endometrial stem cells with high expression of CYR61 onto a collagen scaffold increased angiogenesis and enhanced repair of a full-thickness endometrial injury model in animals 66. According to Park et al., transplantation of perivascular endometrial stem cells can improve implantation defects and poor pregnancy outcomes through HIF1α-dependent angiogenesis in a dose-dependent manner. Additionally, their analysis of the secretome of these stem cells revealed that cyclophilin-A was the primary factor utilized in HIF1α-dependent angiogenesis for cell therapy 67. The location of perivascular stem cells in the perivascular space of the endometrial tissue, as well as their molecular characteristics, are properly illustrated in Fig. 4.

## Various endometrial disorders associated with dysregulation of endometrial stem cells

Endometrial stem cells have been implicated in the pathogenesis of several gynecological disorders, including endometriosis, uterine fibroids, adenomyosis, thin endometrium, and endometrial cancer. For example, recent studies have suggested that endometrial stem cells might contribute to the development and progression of endometriosis by differentiating into endometrial-like cells in ectopic locations 68 and by promoting the establishment of a favorable microenvironment for the survival and growth of endometrial cells in the peritoneal cavity 69. Recent studies have also suggested that endometrial stem cells might contribute to the development and progression of endometrial cancer by acquiring genetic and epigenetic alterations that promote their transformation into malignant cells 70, 71. Overall, endometrial stem cells have attracted intense research interest due to their potential involvement in several diseases and disorders affecting the female reproductive system. Understanding their biology and function might lead to the development of new therapeutic strategies for these endometrial disorders. Therefore, the role of endometrial stem cells in the development of various gynecological disorders has been described.

### Endometriosis

Endometriosis is a widespread and benign gynecological condition that is often accompanied by pelvic pain and infertility. This disorder is characterized by the presence of endometrial-like tissue in ectopic locations within the body, which can undergo significant changes due to menstrual cycle hormones 72, 73. This disease affects about 10% of women of reproductive age 74-76. It can cause severe pain during menstruation, sexual intercourse, and other daily activities 77. Furthermore, a wide range of symptoms associated with endometriosis can make its diagnosis challenging, often leading to misdiagnosis. The current standard for diagnosis is an invasive procedure such as laparoscopy or laparotomy, which can be uncomfortable with risk of complications. In addition to diagnosis, the severity of the disease can also be assessed using a scoring system that considers factors such as lesion size, depth, and location 78. At present, there are no effective curative measures available for endometriosis mainly due to the unclear etiology and pathogenesis of this disease. While the theory of retrograde menstruation is widely accepted as the most plausible explanation for the development of endometriosis 79, 80, it remains a contentious issue within the medical community. Additionally, no current theories provide a complete understanding of the pathogenesis of endometriosis, which further complicates the development of effective treatment options. During menstruation, eutopic endometrial tissue is normally shed and expelled through the cervix. However, in some cases, this tissue can also enter the peritoneal cavity via fallopian tubes, resulting in the development of endometriotic lesions 81. While retrograde menstruation is a contributing factor in the development of endometriosis, it is only one aspect of a much larger and complex picture. In fact, the majority (between 76% to 90%) of women experience retrograde menstruation 82. However, only a small proportion of them develop endometriosis. Therefore, it is clear that other factors play a significant role in the development and progression of this disease.

**Figure 5:**
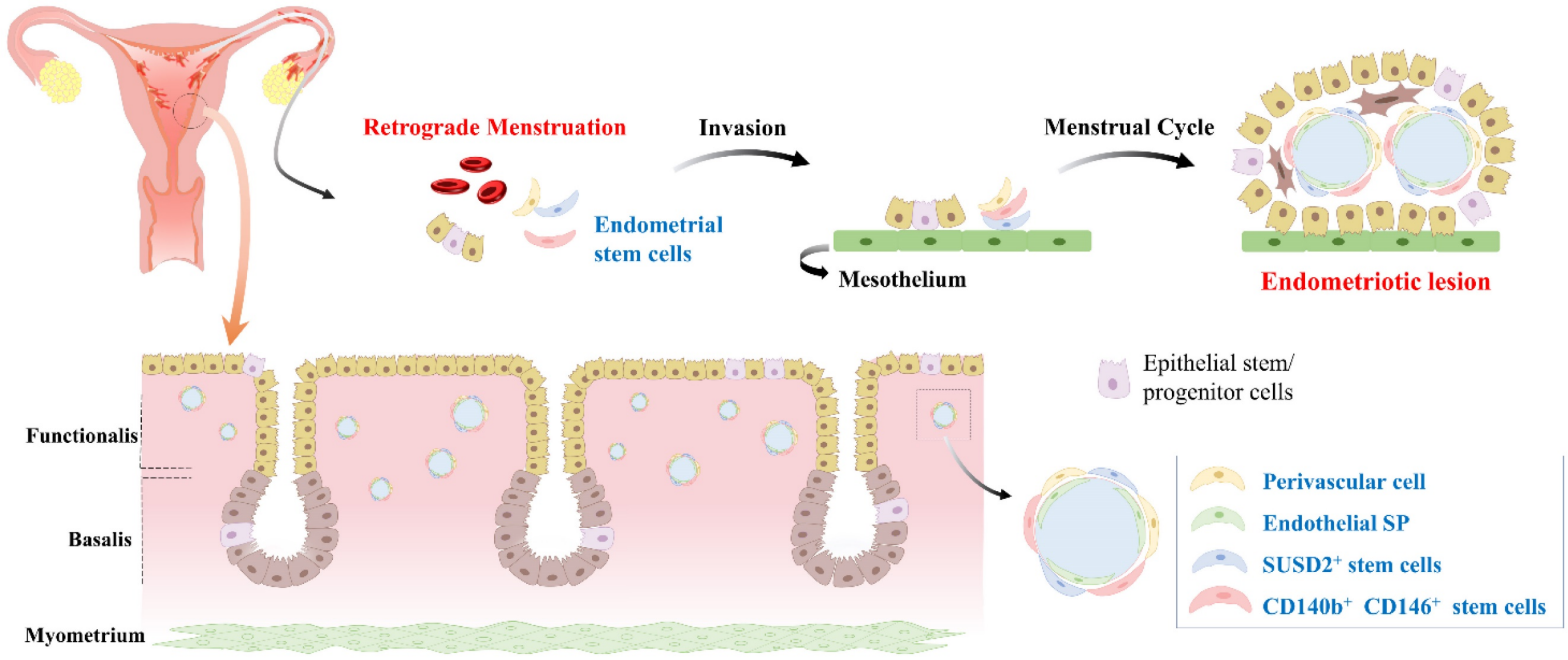
** Development of endometriosis through retrograde menstruation and dissemination of endometrial stem cells within eutopic endometrial tissue.** During menstruation, a portion of the endometrial tissue is expelled from the uterus and flows back through the fallopian tubes into the abdominal cavity, instead of being expelled externally. This retrograde menstruation process is a common occurrence in women, but in some cases, it can lead to the development of endometriosis. During retrograde menstruation, these endometrial stem cells, along with other cellular components, are deposited in different areas of the abdominal cavity, such as the peritoneum, ovaries, and fallopian tubes. Abnormal endometrial stem cells play a pivotal role in the development of endometriosis. These cells exhibit altered molecular profiles, including dysregulated expression of genes involved in cell adhesion, invasion, angiogenesis, and immune response. Abnormal endometrial stem cells have been shown to possess enhanced survival capabilities, resistance to apoptosis, and increased angiogenic potential, facilitating the establishment and growth of endometriotic lesions.

Although the exact molecular mechanisms underlying the development and progression of endometriosis are not fully understood yet, recent research has suggested that endometrial stem cells might play a critical role in this disease. This hypothesis is gaining increasing attention as a potential explanation for the complex and multifactorial pathogenesis of endometriosis 51. One proposed mechanism is that endometrial stem cells can differentiate into endometrial-like cells outside the uterus and contribute to the formation of endometriotic lesions (Fig. 5). Indeed, Moggio et al. have observed that ectopic endometrial stem cells obtained from endometriosis patients exhibit significantly greater proliferation, migration, and angiogenesis capabilities than eutopic endometrial stem cells from the same individual or healthy controls 83. This finding suggests that the aberrant behavior of endometrial stem cells might contribute to the pathogenesis of endometriosis. Uzan et al. have found that expression levels of PTEN, ARID1A, and TNFα in CD73^+^CD90^+^CD105^+^ endometrial stem cells are significantly down-regulated in paired-ectopic samples compared to eutopic endometrium samples 84. This finding suggests that aberrant expression of specific genes might predispose endometrial stem cells to the development of endometriosis. Similarly, Nikoo et al. have revealed higher expression levels of CD9, CD10, and CD29 with increased proliferative potential and migratory capacity of endometrial stem cells from patients with endometriosis compared to those from women without endometriosis 85.

### Uterine fibroids

Uterine leiomyomas or fibroids are hormonally responsive tumors that arise from smooth muscle cells of the myometrium. These tumors are the most common benign uterine neoplasms. They range in size from small, clinically insignificant nodules to large masses that distort the shape and size of the uterus. They are particularly prevalent in women of reproductive age, with up to 70% of women developing fibroids at some point in their lives. While most fibroids are asymptomatic without requiring treatment, approximately 25-50% of affected women experience severe clinical symptoms such as pelvic pain, heavy menstrual bleeding, and infertility 86, 87. These symptoms can significantly impact a woman's quality of life. They might require medical or surgical intervention. Although the exact cause of uterine fibroids remains unclear, estrogen and progesterone are thought to play a role in their development and growth.

To account for the remarkable plasticity of uterine fibroids, it has been suggested that somatic stem cells may exist within the tissue and that mutations or dysregulation of these cells could contribute to the development of uterine fibroids 88, 89. These suggestions are supported by evidence that uterine fibroids are composed of various cell types, including smooth muscle cells, fibroblasts, and extracellular matrix, which can all arise from a common stem cell precursor 88. While the exact cause of uterine fibroids is not fully understood yet, several lines of evidence suggest that endometrial stem cells might play a role in their development. Endometrial stem cells are capable of differentiating into various cell types including smooth muscle cells 90, 91, which are the main component of uterine fibroids. It has been suggested that aberrant differentiation of endometrial stem cells into smooth muscle cells might contribute to the development of uterine fibroids 92, 93. In addition, several studies have reported the presence of stem cell markers in uterine fibroids, indicating possible involvement of endometrial stem cells in their development 94. For example, Mas et al. have successfully isolated stem cells from human fibroids and adjacent myometrium tissues. They identified a specific subpopulation of stem/progenitor cells characterized by expression of Stro-1 and CD44. These cells are present in both myometrial and fibroid tissues 94. They also observed that these Stro-1/CD44 double-positive stem cells had the capacity to differentiate into mesenchymal lineage cell types. Moreover, they were able to demonstrate that these cells had the ability to form myometrial/fibroid-like tissues in an animal model. These findings suggest that the Stro-1/CD44 double-positive stem cell subpopulation could play a significant role in the development and progression of fibroids. Fernung et al. have proposed a hypothesis that normal myometrial stem cells might undergo transformation into tumor-initiating stem cells, leading to the development of uterine fibroids possibly due to somatic mutations in the MED12 gene of unknown origin. They also found that stem cells isolated from human fibroid tissues exhibited differential expression of DNA repair genes associated with DNA double- and single-strand breaks compared to stem cells from adjacent myometrium tissues. These observations suggest that differential DNA repair gene expression in stem cells might contribute to the pathogenesis of uterine fibroids 95. Similarly, Patterson et al. have identified CD146^+^/CD140b^+^ and/or SUSD2^+^ myometrial and fibroid stem-like cells in the perivascular region with a higher colony-forming ability *in vitro* than control cells 96. They also noted that SUSD2^+^ myometrial stem-like cells exhibited greater *in vitro* decidualization potential, whereas SUSD2^+^ fibroid stem-like cells formed larger tumors *in vivo* than control cells 96. These findings suggest that CD146^+^/CD140b^+^ and/or SUSD2^+^ stem-like cells might play a significant role in the pathogenesis of uterine fibroids.

### Adenomyosis

Adenomyosis is a common gynecological disease characterized by infiltration of endometrial glands and stromal cells into the myometrium of the uterus, resulting in formation of nodules or islands. This infiltration is thought to be driven by estrogen, which promotes growth and proliferation of endometrial cells 97, 98. Its resulting symptoms include painful menstruation, pelvic pain, abnormal bleeding, and subsequent infertility 98, 99. Epidemiological studies have reported that the estimated prevalence of adenomyosis ranges from 20% to 35% in females 100. The exact pathophysiology of adenomyosis remains poorly understood. However, several mechanisms have been proposed. One theory suggests that adenomyosis might be caused by abnormal migration of endometrial cells into the myometrium during embryonic development of the uterus 97, 101. Another theory proposes that adenomyosis might be a result of damage to the lining of the uterus, such as that caused by childbirth, surgery, or inflammation 102, 103. Hormonal imbalances, particularly elevated estrogen levels, might also contribute to the development and progression of adenomyosis 104. At the cellular level, the pathogenesis of adenomyosis involves complex interactions among multiple immune cell types. Numbers of macrophages, natural killer cells, and T cells in the endometrial stroma of adenomyosis have been reported to increase significantly compared to those in women without the disease or with mild focal adenomyosis. These immune cells secrete various cytokines, chemokines, and growth factors known to interact with endometrial epithelial and stromal cells, leading to infiltration and survival of endometrial cells in the myometrium 105.

While the exact molecular mechanism involved in adenomyosis development is not fully understood yet, several lines of evidence suggest potential roles of endometrial stem cells. Indeed, Chen et al. have revealed an abnormal upregulation of Musashi-1, an endometrial somatic stem cell marker reflecting their proliferative potential, in the ectopic endometria of patients with adenomyosis, indicating the potential involvement of adult stem/progenitor cells in the development of the disease 106. Kozachenko et al. have reported a similar finding, observing an increased expression of Musashi-1 in adenomyotic foci compared to endometrial cells 107. They further noted that the most intense staining was observed in nodular adenomyosis, particularly in epithelial cells during the secretion phase. These findings suggest that somatic stem/progenitor cells might play a crucial role in the pathogenesis of adenomyosis 107. Lupicka et al. have also discovered that mRNA expression levels of three multipotency markers, namely NANOG, OCT4, and SOX2, are elevated in myometrial cells derived from uteri with adenomyotic lesions compared to those from normal uteri 108. Interestingly, Shilina et al. have conducted a study to investigate characteristics of mesenchymal stem cells derived from a patient with adenomyosis. They compared these patient-derived stem cells to mesenchymal stem cells obtained from healthy donors and found that both cell types have a similar fibroblast-like morphology with the same expression levels of surface markers and adipogenic potential. However, when karyotypes of patient-derived stem cells were analyzed, chromosomal abnormalities were frequently observed, including aneuploidy and nonrandom chromosome breaks, with chromosomes 7 and 11 being affected more often than others. These findings suggest that endometrial stem cells from patients with adenomyosis might have genetic instability, which could contribute to the development and progression of the disease 109.

### Infertility associated thin endometrium

Infertility is a major global health and societal challenge affecting millions of people worldwide, with an increasing incidence over time 110. The inability of the endometrium to support successful embryo implantation is responsible for two-thirds of implantation failures, while embryo quality accounts for the remaining one-third 111. Successful pregnancy is dependent on the development of an embryo within a receptive endometrium of adequate thickness. A thin endometrium is a recognized cause of infertility, recurrent pregnancy loss, and placental abnormalities in clinical practice 112. The proliferative phase of the menstrual cycle is considered critical for determining endometrial thickness. Estrogen promotes the proliferation of endometrial cells and the development of a receptive environment for embryo implantation 113. A thin endometrium is typically defined as having a thickness of less than 7-8 mm in the middle secretory phase. It occurs in up to 8% of infertility cases 114, 115. Although the precise molecular mechanism underlying the involvement of endometrial stem cells in thin endometrium-associated infertility remains incompletely understood, emerging evidence suggests potential implications of endometrial stem cells. Dysfunction or depletion of these stem cells is believed to contribute to the development of a thin endometrium. The regenerative process in the endometrium involves activation of luminal epithelial stem/progenitor cells residing in the basalis layer 5, 116, characterized by their expression of specific markers such as SSEA-1 and SOX9. Throughout the endometrial growth phase, the luminal epithelium progressively ascends, driven by continuous proliferation and differentiation of SSEA-1^+^/SOX9^+^ cells. These cells are crucial for the replenishment and maintenance of the luminal epithelial layer, ensuring its functional integrity and ability to support successful implantation and pregnancy. Activation of residual SSEA-1^+^ luminal epithelial cells during menstruation might play a crucial role in facilitating rapid re-epithelialization during piecemeal shedding of the functionalis layer 44. These SSEA-1^+^ cells possess adhesive and migratory properties, making them well-suited for the regenerative process.

In this context, several studies have been undertaken to restore the thin endometrium utilizing endometrial stem cells. For example, Tersoglio et al. have conducted a comprehensive investigation to assess the impact of endometrial mesenchymal stem cell transplantation on endometrial changes in patients with thin endometrium. Their results revealed a highly significant increase in endometrial thickness following transplantation of endometrial stem cells with high regenerative capacity 117. Similarly, Zhang et al. observed therapeutic effects of human endometrial mesenchymal stem cell transplantation on restoration of injured endometrium. Their findings revealed remarkable improvements in endometrial regeneration and fertility outcomes. At day 7 post-transplantation, the injured endometrium exhibited a significant acceleration in restoration, as evidenced by increased microvessel density and endometrial thickness. Compared to the control group, the stem cell transplanted group exhibited a higher conception rate of 53.57% versus 14.29%, indicating a substantial improvement in successful pregnancies 10.

### Endometrial cancer

Endometrial cancer, a malignancy originating in the uterine lining, has shown a global surge in its incidence. It is poised to become a substantial cause of mortality in women. The prevalence of endometrial carcinoma continues to grow, affecting approximately 62,000 women each year in the United States alone 118. While majority of endometrial cancers are detected at early and treatable stages, management of advanced-stage endometrial cancers presents significant challenges. Despite its relatively favorable 5-year survival rate (reaching up to 80% in most patients), its prognosis becomes grim once patients experience disease recurrence and progress to terminal stage at a rapid pace 119. Endometrial cancer encompasses several distinct subtypes, with the most prevalent being endometrioid adenocarcinoma (EAC) and serous cystadenocarcinoma (SCC). These subtypes differ in terms of microscopic characteristics, metastatic patterns, risk factors, and prognosis 120, 121. Notably, endometrial cancer exhibits significant intra- and inter-tumoral heterogeneity, reflecting diverse molecular and cellular features within and between individual tumors 122.

**Figure 6:**
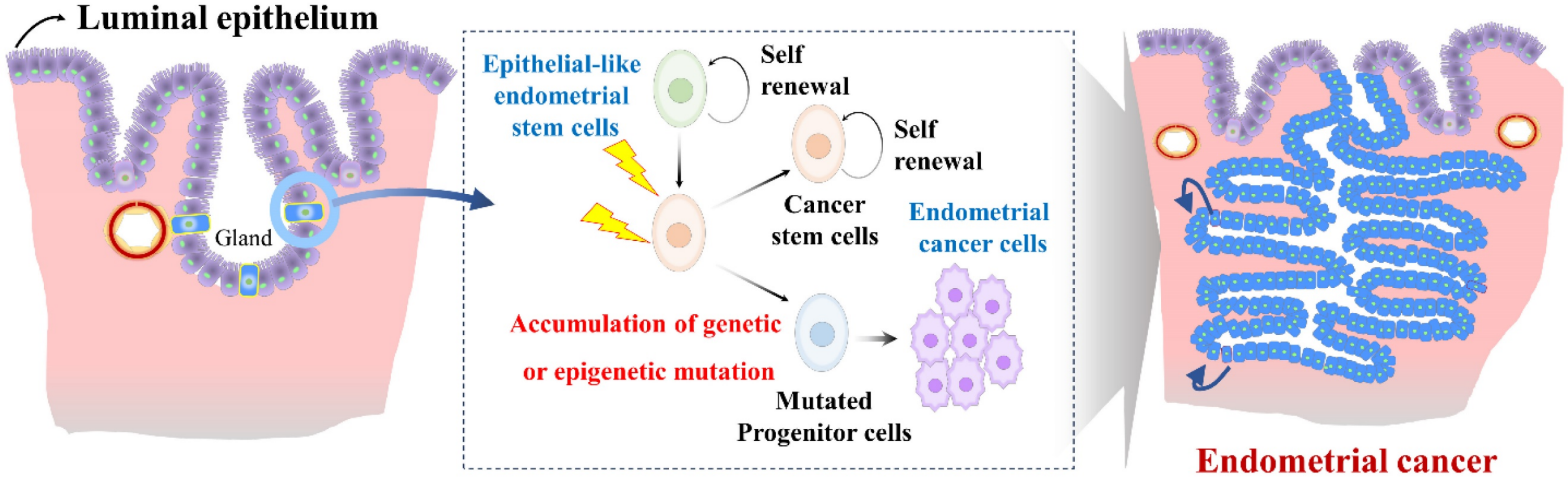
** Role of abnormal endometrial stem cells with accumulated mutations in the development of endometrial cancer.** Abnormal endometrial stem cells exhibit characteristics such as increased self-renewal capacity, altered differentiation potential, and resistance to apoptosis, making them prime candidates for driving their transformation into malignant endometrial cancer cells, resulting in invasive carcinoma. These mutations may affect crucial genes involved in cell cycle regulation, DNA repair, apoptosis, and cellular signaling pathways. Examples of commonly mutated genes in endometrial cancer include Axin2, LCN2, Musashi-1, SAA1/2, SIX1, WNT/β-catenin signaling.

Endometrial stem cells might be involved in the development of endometrial cancer 123 as they show increased proliferative capacity and genetic instability in some cases. Epithelial-like endometrial stem cells have been proposed to play a role in the development and progression of endometrial cancer through several mechanisms. One proposed mechanism is that Epithelial-like endometrial stem cells might accumulate genetic mutations that lead to transformation of normal endometrial stem cells into cancer cells (Fig. 6). For example, Syed et al. have proposed a hypothesis suggesting the presence of Wnt-responsive stem/progenitor cells within endometrial glands. They identified Axin2, a well-known Wnt reporter gene, as a biomarker for epithelial-like stem/progenitor cells residing in the endometrial glands 124. These Axin2-expressing cells play a crucial role in epithelial regeneration *in vivo* and contribute to the development of endometrial organoids *in vitro*. Ablation of Axin2^+^ stem/progenitor cells lead to severe impairment of endometrial homeostasis and compromises its regenerative capacity. Remarkably, during oncogenic transformation, these Axin2^+^ cells are implicated in the development of endometrial carcinogenesis, highlighting significant involvement of tissue resident stem cells in the pathogenesis of endometrial cancer 124. Similarly, aberrant expression of Musashi-1, a protein involved in stem cell maintenance and multipotency, has been observed in primary endometrial cancer tissues. *In vitro* studies have shown that silencing of Musashi-1 leads to a significant decrease in cell proliferation and radioresistance of endometrial cancer 125. Similarly, *in vivo* experiments have confirmed the anti-proliferative effect of Musashi-1 knockdown, with tumors being approximately 40% smaller in size compared to control tumors 125. These findings highlight the potential involvement of tissue resident stem cells in the development and progression of endometrial cancer.

Furthermore, the transcription factor Sineoculis homeobox homolog 1 (SIX1), part of the SIX family of homeoproteins, exhibits elevated expression during embryogenesis, contributing to the expansion and survival of progenitor cells 126, 127. In adult tissues, the expression of SIX1 is typically low, and any abnormal expression of the SIX1 gene in these tissues could potentially play a role in the development of cancer. 128. Previous studies have shown that SIX1 is excessively expressed in various human cancers, including breast 129, cervical 130, and ovarian 131 cancers, and this overexpression is linked to lower survival rates in patients. Xin et al observed that overexpression of SIX1 in endometrial carcinoma is believed to enhance the growth of cancer cells, potentially via pathways mediated by ERK and AKT. 132. Notably, Suen et al. have demonstrated the significance of SIX1 in normal endometrial epithelial differentiation. They discovered that CK14^+^/18^+^ endometrial subpopulations could function as cancer progenitor cells and that SIX1 played a critical role in delaying synthetic estrogen diethylstilbestrol-induced endometrial carcinogenesis by promoting basal differentiation of CK14^+^/18^+^ cells 133. These findings provide insights into the complex interplay between SIX1, endometrial differentiation, and endometrial carcinogenesis.

## Analyzing endometrial stem cell dynamics in health and disease through single-cell techniques

Endometrial stem cells assume a crucial function in the regeneration and repair of the endometrium by undergoing differentiation into diverse cell types within the endometrial tissue. The occurrence and accumulation of mutations within endometrial stem cells is considered a pivotal element in the onset and advancement of diverse endometrial diseases, such as infertility linked to a thin endometrium, endometrial cancers, and endometriosis 7. Nevertheless, a comprehensive understanding of their role in endometrial disorders, including endometriosis, endometrial cancer, and infertility, remains elusive. Conventional bulk sequencing techniques face constraints in comprehensively capturing the heterogeneity and complexity inherent in populations of endometrial stem cells. To address these constraints, recent advancements in single-cell analysis methodologies, such as single-cell ATAC sequencing (scATAC-Seq), single-cell RNA sequencing (scRNA-Seq), and spatial transcriptomics, have surfaced as invaluable tools for investigating endometrial stem cells. For instance, Wang et al. conducted an analysis of transcriptomic alterations within the functionalis layer of the endometrial tissue, a region that experiences cyclical shedding and regeneration during the menstrual cycle. They concentrated on examining dynamic changes in gene expression at the individual cell level within both epithelial and stromal cellular components 134. They noted a significant increase in the expression of CXCL14, GPX3, and PAEP within the epithelial cell subpopulations. In addition, Ren et al. explored the dynamic alterations in diverse cellular components within the endometrial tissue as it transitions from a normal state to endometrial cancer. Their investigation yielded valuable insights into the cellular origins of endometrial cancer and identified certain cell populations linked to the tumor development through the application of scRNA-Seq. 135. Through their investigation, they determined that the origin of endometrial cancer could be traced back to epithelial-like cells rather than stromal-like cells. In a more detailed examination, they recognized unciliated glandular epithelial cell types as cellular origin of endometrial cancer 135. They also detected a unique subset of cells potentially integral to tumor development. These cells were distinguished by their enhanced expressions of the LCN2 and SAA1/2. Furthermore, Vrljicak et al. examined chromatin accessibility patterns in both undifferentiated and differentiated states of endometrial stem cells during decidualization process using ATAC-Seq. They observed a significant decrease in chromatin accessibility throughout the decidualization process of endometrial stem cells 136. This diminished chromatin accessibility was particularly linked to the loss of binding motifs for specific transcription factors (TFs) known to be downregulated during decidualization process. Notably, ETS Proto-Oncogene 1 (ETS1), Runt-related transcription factors 1 and 2 (RUNX1 and RUNX2), and SRY-box 12 (SOX12) were identified as TFs with reduced binding motifs 136. Yu et al. have also employed a hybrid approach, combining scRNA-Seq datasets with spatial transcriptomics data, to explore the cellular interactions and molecular characteristics of various cell components within endometrial cancer tissue 137. They noted that two subpopulations of epithelial cell types, specifically lymphatic endothelial-like cells and blood endothelial-like cells, displayed a more aggressive morphology. This heightened malignancy could potentially be attributed to the activation of the MK signaling via MDL-NCL pathways. 137.

## Future prospective and conclusions

This review provides a comprehensive overview of the dynamic properties of the human endometrium and the roles of various types of endometrial stem cell subpopulations during endometrial regeneration. It is widely recognized that tissue-resident stem/progenitor cells exist within the basal layer of endometrial tissue. Enhanced understanding of their roles offers new perspectives on the remarkable regenerative capabilities displayed by human endometrial tissue, both during tissue injury or the normal menstrual cycle. In comparing endometrial stem cells to other stem cell types, we emphasize their potential advantages in proliferation and differentiation, particularly for uterine-related therapies. While reports suggest that uterine stem cells may surpass bone marrow and adipose-derived stem cells in proliferative and multilineage differentiation capacities, these outcomes can vary based on factors like the donor's condition, culture environments, and *in vitro* passages. This variability underscores the need for more detailed research to fully understand and leverage the unique properties of endometrial stem cells in regenerative medicine. Furthermore, this understanding offers novel perspectives on diverse diseases linked to endometrial stem cells, such as endometrial cancer, endometriosis, and infertility, thus presenting potential therapeutic strategies that target abnormal or dysregulated endometrial stem cells populations. However, further comprehensive investigations are imperative to elucidate the precise genetic and non-genetic mutations responsible for inducing abnormalities in normal endometrial stem cells. Additionally, understanding the impact of these aberrant endometrial stem cells on the pathogenesis of diverse female uterine-related diseases requires deeper exploration. Specifically, the growth and differentiation of endometrial stem cells are significantly modulated by paracrine signaling mediated by diverse growth factors, alongside key hormones like estrogen and progesterone, which dynamically fluctuate throughout the menstrual cycle. Further research should aim to unravel the complex interplay of these signals and their impact on endometrial stem cell fate decisions. Additionally, harnessing the regenerative potential of endometrial stem cells might hold promise in improving fertility outcomes in women with endometrial disorders.
